# Gemcitabine Plus Anlotinib Is Effective and Safe Compared to Gemcitabine Plus Docetaxel in Advanced Soft Tissue Sarcoma

**DOI:** 10.3389/fonc.2022.922127

**Published:** 2022-07-13

**Authors:** Zhiyong Liu, Xin Wang, Jiaqiang Wang, Peng Zhang, Chao Li, Bangmin Wang, Guancong Liu, Weitao Yao

**Affiliations:** Department of Orthopedics, The Affiliated Cancer Hospital of Zhengzhou University and Henan Cancer Hospital, Zhengzhou, China

**Keywords:** efficacy, safety, anlotinib, gemcitabine, docetatxel, soft tissue sarcoma (STS)

## Abstract

**Objective:**

The aim of this study is to compare gemcitabine (G) plus docetaxel (D) versus G plus anlotinib (A) for advanced soft tissue sarcoma (STS).

**Methods:**

We retrospectively investigated 122 patients with locally advanced or metastatic STS who were treated with either G+D or G+A between July 2016 and October 2021 and compared the efficacy and toxicity of G+D and G+A. The primary endpoints were median progression-free survival (PFS) and the proportion of patients with grade ≥3 adverse events. We also analyzed differences in the clinical efficacy of G+D and G+A in leiomyosarcoma, and the differences in the clinical efficacy of G+D and G+A as first-line therapy.

**Results:**

Overall, 122 patients were included (81 patients receiving G+D and 41 patients receiving G+A) with a median age of 55 years. The main histological types are leiomyosarcoma, undifferentiated pleomorphic sarcoma, and liposarcoma. After a median follow-up of 25 months, PFS did not differ between patients treated with G+D and those treated with G+A (median PFS: 5.8 months and 6.8 months, *p* = 0.39), and overall survival (OS) was similar (median OS: 14.7 vs. 13.3 months, *p* = 0.75) with a similar objective response rate (18.5% vs. 14.6%, *p* = 0.17), whereas the proportion of patients with grade ≥3 adverse events treated with G+D was significantly higher than those treated with G+A (68% vs. 44%, *p* < 0.05). Subgroup analysis of leiomyosarcoma patients (47.5% of the patients) and first-line treatment patients (46.7% of the patients) shows that PFS was not significantly different between the two groups (LMS: median PFS: 6.5 months vs. 7.5 months, *p* = 0.08; first-line treatment: median PFS: 6.2 months vs. 7.1 months, *p* = 0.51).

**Conclusion:**

Compared with gemcitabine plus docetaxel for advanced STS, gemcitabine plus anlotinib achieved a similar response rate on median PFS and OS, but lower toxicity. These results suggest that gemcitabine plus anlotinib may be an effective and safe strategy for advanced STS.

## Introduction

Soft tissue sarcoma (STS) comprises rare and profound heterogeneous tumors of mesenchymal origin, with more than 50 different histological subtypes and an incidence of 10/1 million (1–3). Over 50% of STS originates in the trunk and limbs. Despite radical surgical excision with or without radiotherapy, approximately one-third of patients will eventually experience recurrence of locally unresectable or metastatic disease that threatens organ function and life (4). In the locally advanced or metastatic disease setting, patients have a poor prognosis, with a reported median overall survival (OS) of 12–20 months after diagnosis (5). The treatment of advanced disease with systemic therapy is limited, and cytotoxic chemotherapy has a prominent role in standard treatments consisting of doxorubicin monotherapy or doxorubicin plus ifosfamide (6). However, the cumulative cardiotoxicity of doxorubin increases significantly at doses above 450 mg/m^2^, which limits the sustainability of clinical application (7). Taking into account the heterogeneous histomorphology and behavior of the disease, second-line treatment regimens are usually individualized based on histological subtypes and clinical behaviors (1), such as high-dose cyclophosphamide for synovial sarcoma (8), eribulin for liposarcoma (9), and tyrosine kinase inhibitors for non-adipogenic STS including pazopanib (10), regorafenib (11), and anlotinib (12). Apart from these drugs, gemcitabine (G) and/or docetaxel (D) is another widely used regimen for most patients with advanced STS (6).

Although G+D has shown moderate antitumor activity in advanced STS, the role of D remains controversial because multiple studies have yielded conflicting results with the exception of angiosarcomas (13). A Phase II study (14) published in 2002 including 119 patients with metastatic STS treated as one to four lines of chemotherapy showed that, compared with G monotherapy, G+D yielded statistically significant benefits in response rate (16% vs. 8%), median progression-free survival (PFS) (6.2 months vs. 3 months), and median OS (17.9 months vs. 11.5 months), but increased toxicity, where the toxicity results in at least one dose reduction in 46% of patients. In subtype analysis, the combination therapy could lead to superior survival in leiomyosarcoma (LMS) and undifferentiated pleomorphic sarcoma (UPS) compared with other subtypes. Moreover, a preclinical study showed that G followed by D is synergistic, and otherwise antagonistic (15). Hence, the current G+D regimen evolved into a most effective method for the treatment of advanced STS. However, several Phase II and retrospective studies showed that G+D showed moderate clinical efficacy similar to that of G alone, but with more adverse events (AEs). In 2012, the French Sarcoma Group conducted a randomized stratified Phase II study (16), which examined the efficacy and toxicity of G+D and G in 90 patients with metastatic or inoperable LMS (uterine and non-uterine) who had failed first-line therapy. For patients with non-uterine LMS, ORR was 5% and 14% for G+D and G treatment, and median PFS was 3.8 and 6.3 months, respectively. For patients with uterine LMS, G+D and G had similar effects, with ORR of 24% and 19% and median PFS of 4.7 and 5.5 months, respectively. However, less toxicity was observed in patients treated with G alone. Such results suggested that gemcitabine-based therapy is a treatment option for patients who have failed anthracycline therapy.

Chemotherapy and anti-angiogenesis play a crucial role in advanced malignant tumors. Increasing studies (17–20) have shown that anti-angiogenesis in combination with chemotherapy yielded synergetic effects in several malignancies including breast cancer, lung cancer, and sarcomas. Preclinical studies (21, 22) showed that anti-angiogenesis therapy not only induced tumor cell apoptosis but also induced tumor vascular normalization, improved hypoxia, and increased the delivery of chemotherapy drugs. In the ALTER0203 trial (12), anlotinib demonstrated superior ORR (10.13% vs. 1.33%) and median PFS (6.27 months vs. 1.47 months) over placebo in advanced STS after the failure of standard chemotherapy. Based on the above results, it was approved in China as a second-line treatment drug for advanced STS except for alveolar STS and clear cell sarcoma, in June 2019. Preclinical studies (23, 24) showed that anlotinib played a crucial part in tumor cell proliferation and migration, as well as angiogenesis, though selectively targeting VEGFR, PDGFR, FGFR, c- kit, and MET. The IC_50_ value was 0.2 nmol/L for VEGFR2, 0.7 nmol/L for VEGFR3, 8.7 nmol/L for PDFGFRβ, and 11.7 nmol/L for FGFR1. These data indicate that anlotinib has the potential to simultaneously inhibit phosphorylation of important tyrosine kinases and their downstream signaling pathways. Considering the poor outcome of advanced STS and the potential synergistic effect of gemcitabine and anlotinib (25), G+A has been used as a novel treatment for patients after failure of anthracycline-based regimen since the widespread use of anlotinib in China.

In this study, the efficacy and toxicity of G+D and G+A were retrospectively investigated, aiming to evaluate the feasibility of G+A as a novel treatment option for patients with locally advanced or metastatic STS who had failed adriamycin-based therapy.

## Materials and Methods

### Study Design

Patients with locally advanced or metastatic STS were retrospectively collected between July 2016 and October 2021 at Henan Cancer Hospital, one of the largest hospitals for cancer patients in a province of about 100 million people. Medical records of the patients treated with G+D between July 2016 and October 2021 were reviewed, including patient characteristics, treatment regimens, imaging data, AEs, and survival data, and those of similar patients treated with G+A between July 2019 and October 2021. Partial survival data were followed up by telephone.

The inclusion criteria are as follows: (1) 18–70 years old; (2) histologically proven advanced STS (locally advanced or metastatic); (3) prior treatment with anthracyclines, and adjuvant therapy received within 1 year by patients with the recurrent disease was considered first-line therapy; (4) with measurable lesions according to the Response Evaluation Criteria in Solid Tumors (RECIST) version 1.1; (5) an Eastern Cooperative Oncology Group (ECOG) performance status of 0–2; (6) with target lesions not suitable for complete surgical resection or radiotherapy; (7) adequate blood, renal, liver, and cardiac parameters; (8) available clinical data including medical history, treatment, and survival data; and (9) treated with G+D or G+A.

Exclusion criteria are as follows: (1) patients given other anti-tumor therapies during treatment including chemotherapy, radiotherapy, targeted therapy, surgery, and immunotherapy; (2) patients receiving G+D with a history of prior treatment with G and/or D within 6 months, and patients receiving G+A with a history of prior treatment with G and/or targeted agents within 6 months; (3) risk of active bleeding; (4) treatment interruption lasted more than 6 weeks.

Approval of this study was achieved from the institutional review board of Henan Cancer Hospital according to the Declaration of Helsinki. Written informed consent for treatment from each patient was obtained before either regimen.

### Therapeutic Regimen

For patients treated with G+D, intravenous G 1,000 mg/m^2^ was administered on days 1 and 8 and D 75 mg/m^2^ was given on day 8 every 3 weeks. For G+A, G was administered at the same dose and schedule as the G+D regimen, and A was administered at a dose of 12 mg for 8–21 days every 3 weeks. The treatment schedule was formulated by the patient’s physician and the dose of the respective drugs was reduced according to the toxicities. Granulocyte colony-stimulating factor or pegylated recombinant human granulocyte colony-stimulating factor was routinely given according to AEs. Patients were treated until the disease progressed or intolerable AEs occurred or they refused treatment. Multiple-dose reductions of regimens allowed were observed on an individual basis according to the toxicity. Generally, gemcitabine doses were reduced from 1000 mg/m^2^ to 850 mg/m^2^ and 723 mg/m^2^, docetaxel doses were reduced from 75 mg/m^2^ to 64 mg/m^2^ and 54 mg/m^2^, and anlotinib doses were reduced from 12 mg to 10 mg and 8 mg. Patients were informed of the potential efficacy and toxicity of the regimens before treatment initiation.

### Study Assessments

The primary endpoints were median PFS and the proportion of patients with grade ≥3 AEs in two groups. Secondary endpoints were ORR, OS, and AEs. PFS was the interval from the start of either G+D or G+A to the time of the first occurrence of disease progression, death, or the last follow-up. OS was the interval from the beginning of treatment to the date of death or the last follow-up. The proportion of patients with AEs was the ratio of the number of patients who underwent AEs to the total number of patients. Treatment response was determined by CT scan, MRI scan, or both performed at baseline and then every 1.5–2 months. Tumor response was evaluated according to RECIST 1.1, including complete response (CR), partial response (PR), stable disease (SD), and progressive disease (PD). ORR was defined as the proportion of patients who gained a CR or PR. AEs were collected and evaluated based on the Common Terminology Criteria for Adverse Events (CTCAE) v4.0.

### Statistical Analysis

Descriptive statistics and frequency were used to describe characteristics of patients and sarcomas. The differences in response rate, AEs, and other classification variables between the groups were analyzed by the Chi-square test or Fisher’s exact test. Kaplan–Meier curve was plotted to estimate PFS and OS with 2-sided 95% confidence intervals (CIs), and log-rank test was used to calculate survival distributions. Bilateral *p* < 0.05 was considered statistically significant, and statistical analysis was conducted using GraphPad Prism version 9.0.1.

## Results

### Demographics

From July 2016 to October 2021, 122 patients with locally advanced or metastatic STS who met the inclusion and exclusion criteria were retrospectively identified. Patient characteristics at baseline between two treatment groups were comparable and displayed in **Table 1**. Of 122 patients, 81 patients received G+D and 41 received G+A. The median (range) age was 53 (18–70) and 57 (20–70) years, 32 (39.5%) and 15 (36.6%) patients were men, and 73 (90.1%) and 35 (85.4%) patients had an ECOG PS of 0/1 in the G+D and G+A groups, respectively. The major histological subtypes in the G+D and G+A groups were represented: LMS (38 [46.9%] and 20 [48.7%] patients), UPS (11 [13.6%] and 6 [14.6%] patients), and liposarcoma (LPS) (12 [14.8%] and 4 [9.8%] patients). Seventy-three (90.3%) and 36 (87.8%) patients had received operations, and 30 (37%) and 13 (34.1%) patients had received radiotherapy in the G+D and G+A groups, respectively. In total, more than 70% of patients received either the regimen as a first or second line. Thirty-eight (31.1%) patients received anthracyclines and 84 (68.9%) received anthracyclines plus ifosfamide. Rare sarcoma subtypes with a frequency of 1–4 were defined as others. No significant differences were observed between the two groups in age, sex, histological type, and other characteristics at baseline.

**Table 1:** Demographics of patients.

Characteristics	Overall, *n* (%)	Gemcitabine+Docetaxel, *n* (%)	Gemcitabine+Anlotinib, *n* (%)	*p*-Value
**No. of patients**	122	81	41	
**Age, median (range), years**	55 (18–70)	53 (18–70)	57 (20–70)	0.35
**Sex**				
** Female**	75 (61.5%)	49 (60.5%)	26 (63.4%)	0.75
** Male**	47 (38.5%)	32 (39.5%)	15 (36.6%)	
**ECOG**				
** 0/1**	108 (88.6%)	73 (90.1%)	35 (85.4%)	0.63
** 2**	14 (11.5%)	8 (9.9%)	6 (14.6%)	
**Stage**				
** Metastatic**	94 (77%)	65 (80.5%)	29 (70.7%)	0.24
** Locally advanced**	28 (23%)	16 (19.5)	12 (29.3)	
**Histological types**				
** Leiomyosarcoma**	58 (47.5%)	38 (46.9%)	20 (48.8%)	0.85
** Non-leiomyosarcoma**	64 (52.5%)	43 (53.1%)	21 (51.3%)	
** Undifferentiated pleomorphic sarcoma**	17 (13.9%)	11 (13.6%)	6 (14.6%)	0.74
** Liposarcoma**	16 (13.1%)	12 (14.8%)	4 (9.8%)	
** Others**	31 (25.4%)	20 (24.7%)	11 (26.8%)	
**Prior therapies received**				
** Radiotherapy**	43 (35.2%)	30 (37%)	13 (34.1%)	0.60
** Surgery**	109 (89.3%)	73 (90.3%)	36 (87.8%)	0.94
** Chemotherapy**	122 (100%)	81 (100%)	41 (100%)	
** Anthracyclines**	38 (31.1%)	24 (29.6%)	14 (34.1%)	0.61
** Anthracyclines+Ifosfamide**	84 (68.9%)	57 (70.4%)	27 (65.9%)	
**No. of lines of prior chemotherapy**				
**Neoadjuvant chemotherapy**	57 (46.7%)	37 (45.7%)	20 (48.8%)	0.58
**1**	36 (29.5%)	23 (28.4%)	13 (31.7%)	
**≥2**	28 (23%)	21 (25.9%)	7 (17.1%)	

ECOG, Eastern Cooperative Oncology Group; Others, Synovial sarcoma, Undifferentiated rhabdomyosarcoma, Angiosarcoma, Epithelioid sarcoma, Fibrosarcoma, and Malignant peripheral nerve sheath tumor.

### Treatment Administered

The median of 6 (1–8) cycles of G and the median of 4 (1–7) cycles of D were given in the G+D regimen; G was administered at 1,000 mg/m^2^ in 73 (90.1%) patients, 850 mg/m^2^ in 6 (7.4%) patients, and 723 mg/m^2^ in 2 (2.5%) patients as the beginning dose. D was administered at 75 mg/m^2^ in 67 (82.7%) patients, 64 mg/m^2^ in 11 (13.6%) patients, and 54 mg/m^2^ in 3 (3.7%) patients as the beginning dose. Some patients were given day 1 G, but they were not given day 8 G and/or D; 15% and 30% of patients experienced dose reduction of G and D. While the median of 6 (range 1–8) cycles of G and the median of 6 (1–35) cycles of A were given in the G+A regimen, 34% of patients were treated with G+A followed by A alone because of the poor tolerability of G; 19.5% and 15% of patients experienced dose reductions of G and A, respectively. The main reasons for dose reduction in the two regimens were febrile neutropenia, other hematological toxicities, and non-hematological toxicities (**Tables 2**, **Table 3**).

**Table 2:** Treated details.

	Gemcitabine+Docetaxel	Gemcitabine+Anlotinib
**Median no. of cycles of G (range)**	6 (1–8)	6 (1–8)
**Median no. of cycles of D (range)**	4 (1–5)	
**Median no. of cycles of A (range)**		7 (1–35)
**Dose reduction of G+D, *n* (%)**	25 (30.9%)	9 (22%)
**Dose reduction of G, *n*** (%)	12 (14.8%)	8 (19.5%)
**Dose reduction of D, *n* (%)**	24 (29.6%)	
**Dose reduction of A, *n* (%)**		6 (14.6%)
**Treatment completed**		
**Disease progression, *n* (%)**	57 (70.4%)	31 (75.6%)
**Toxicity, *n* (%)**	20 (24.7%)	6 (14.6%)
**Other reasons, *n* (%)**	4 (4.9%)	4 (9.8%)

G, Gemcitabine; D, Docetaxel; A, Anlotinib; Other reasons, refusal due to not toxicity.

**Table 3:** Dose summary.

Gemcitabine+Docetaxel, *n* (%)		Gemcitabine+Anlotinib, *n* (%)	
Gemcitabine		Gemcitabine	
**No. of cycles**	400 (100%)	No. of cycles	236 (100%)
**Day 1 dose**		Day 1 dose	
**1,000 mg/m^2^**	342 (85.5%)	1,000 mg/m^2^	223 (94.5%)
**850 mg/m^2^**	38 (9.5%)	850 mg/m^2^	10 (4.2%)
**723 mg/m^2^**	20 (5%)	723 mg/m^2^	3 (1.2%)
**Any dose reduction/Lowest dose administered**	12 (14.8%)	Any dose reduction/Lowest dose administered	8 (19.5%)
**850 mg/m^2^**	12 (37.5%)	850 mg/m^2^	15 (62.5%)
**723 mg/m^2^**	16 (50%)	723 mg/m^2^	6 (25%)
**615 mg/m^2^**	4 (12.5%)	615 mg/m^2^	3 (12.5%)
**Docetaxel** **No. of cycles (day 1 dose)**	360 (100%)	Anlotinib No. of cycles (day 1 dose)	400 (100%)
**75 mg/m^2^**	318 (88.3%)	12 mg	390 (97.5%)
**64 mg/m^2^**	36 (10%)	10 mg	10 (2.5%)
**54 mg/m^2^**	6 (1.7%)	8 mg	0 (%)
**Any dose reduction/Lowest dose administered**	24 (30%)	Any dose reduction/Lowest dose administered	6 (14.6%)
**64 mg/m^2^**	24	10 mg	13
**54 mg/m^2^**	16	8 mg	6
**46 mg/m^2^**	10		

### Treatment Responses and survival

Totally, the median follow-up time was 25 months (range, 3–38 months). At the time of the analysis, 117 patients (96%) have completed the treatment, 21 patients have achieved a CR or PR and 62 patients have obtained SD, yielding an ORR and disease control rate of 17.2% and 68%, respectively. The median PFS and OS were 6.3 months (95% CI 6.0–8.4) and 14.3 months (95% CI 14.1–17.4), respectively (**Table 4**). The common reasons for treatment discontinuations were PD in 88 (72.1%) patients, toxicity in 26 (21.3%) patients, and other reasons in 8 (6.6%) patients (**Table 2**).

**Table 4:** Efficacy endpoints.

Endpoints	Two treatment regimens	Gemcitabine+Docetaxel	Gemcitabine+Anlotinib	*p*-Value
**No. of patients**	122	81	41	
**Median PFS (95% CI), months**	6.3 (6.0–8.4)	5.8 (6.0–8.4)	6.8 (6.2–9.2)	0.39
**Median OS (95% CI), months**	14.3 (14.1–17.4)	14.7 (13.9–17.9)	13.3 (12.8–18.0)	0.75
**Best response, *n*** (%)				
**Complete response**	1 (0.8%)	1 (1.2%)	0	NA
**Partial response**	15 (12.3%)	14 (17.3%)	6 (14.6%)	0.47
**Stable disease**	62 (50.8%)	39 (48.1%)	23 (56.1%)	0.22
**Progressive disease**	39 (32%)	27 (33.3%)	12 (29.3%)	0.43
**Objective response rate**	21 (17.2%)	15 (18.5%)	6 (14.6%)	0.17
**Disease control rate**	83 (68%)	54 (66.7%)	29 (70.7%)	0.58

95% CI, 95% confidence interval; PFS, progression-free survival; OS, overall survival.

**Figure 1:**
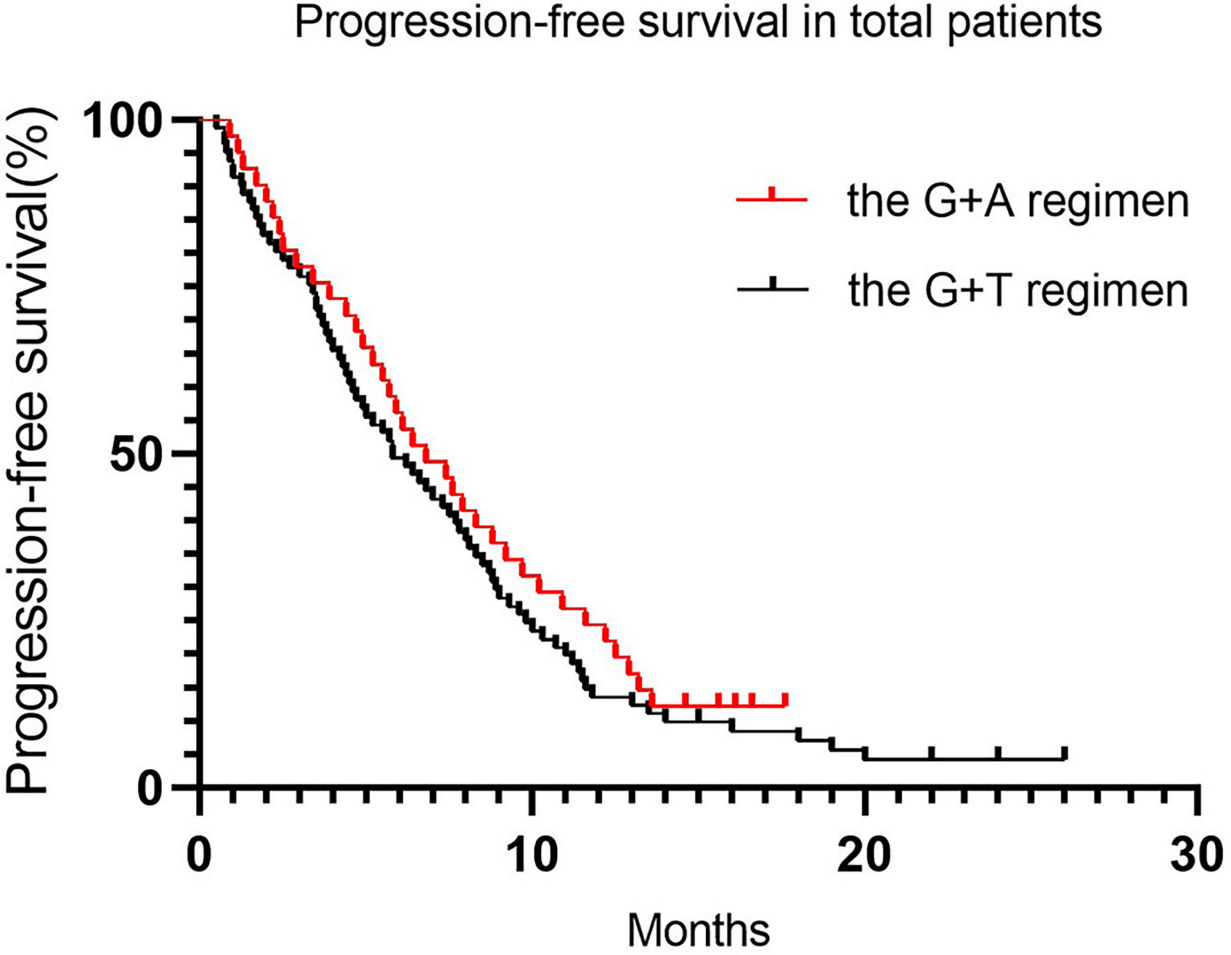
Progression-free survival in total patients.

**Figure 2:**
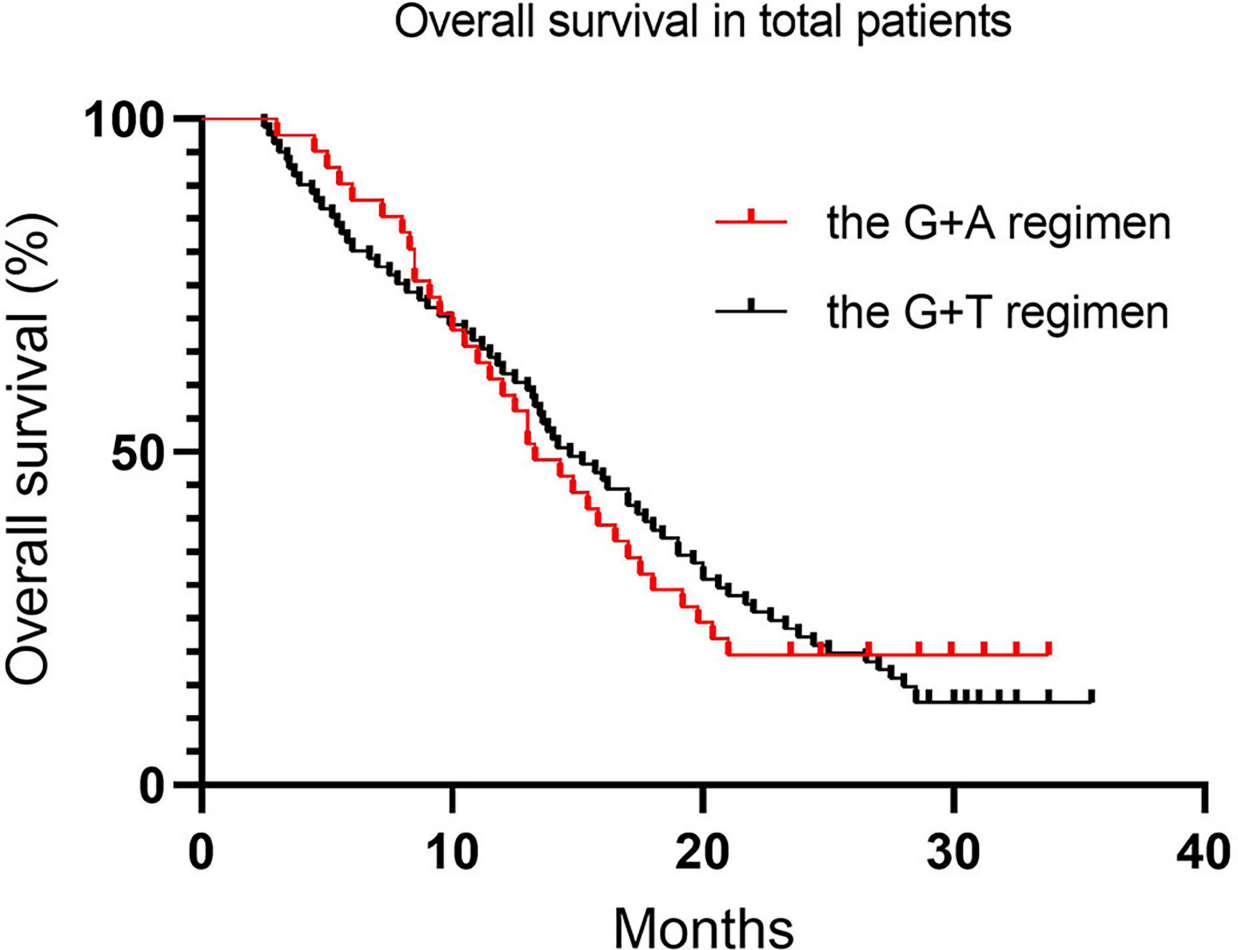
Overall survival in total patients.

Regarding differences in efficacy in total patients, no statistically significant difference in PFS was detected between the treatment groups, with a median PFS of 5.8 months (95% CI 6.0–8.4) vs. 6.8 months (95% CI 6.2–9.2) for the G+D and G+A groups, respectively (**Table 4** and **Figure 1**, *p* = 0.39). Similarly, no statistically significant difference in OS was detected between the treatment groups, with a median OS of 14.7 months (95% CI 13.9–17.9) vs. 13.3 months for the G+D and G+A groups (**Table 4** and **Figure 2**, *p* = 0.75). Response rates were similar between the G+D and the G+A groups, with an ORR of 18.5% (15/81) vs. 14.6% (6/41) (**Table 4**, *p* = 0.17) and a DCR of 66.7% (54/81) vs. 70.7% (29/41) (**Table 4**, *p* = 0.75), respectively.

**Figure 3:**
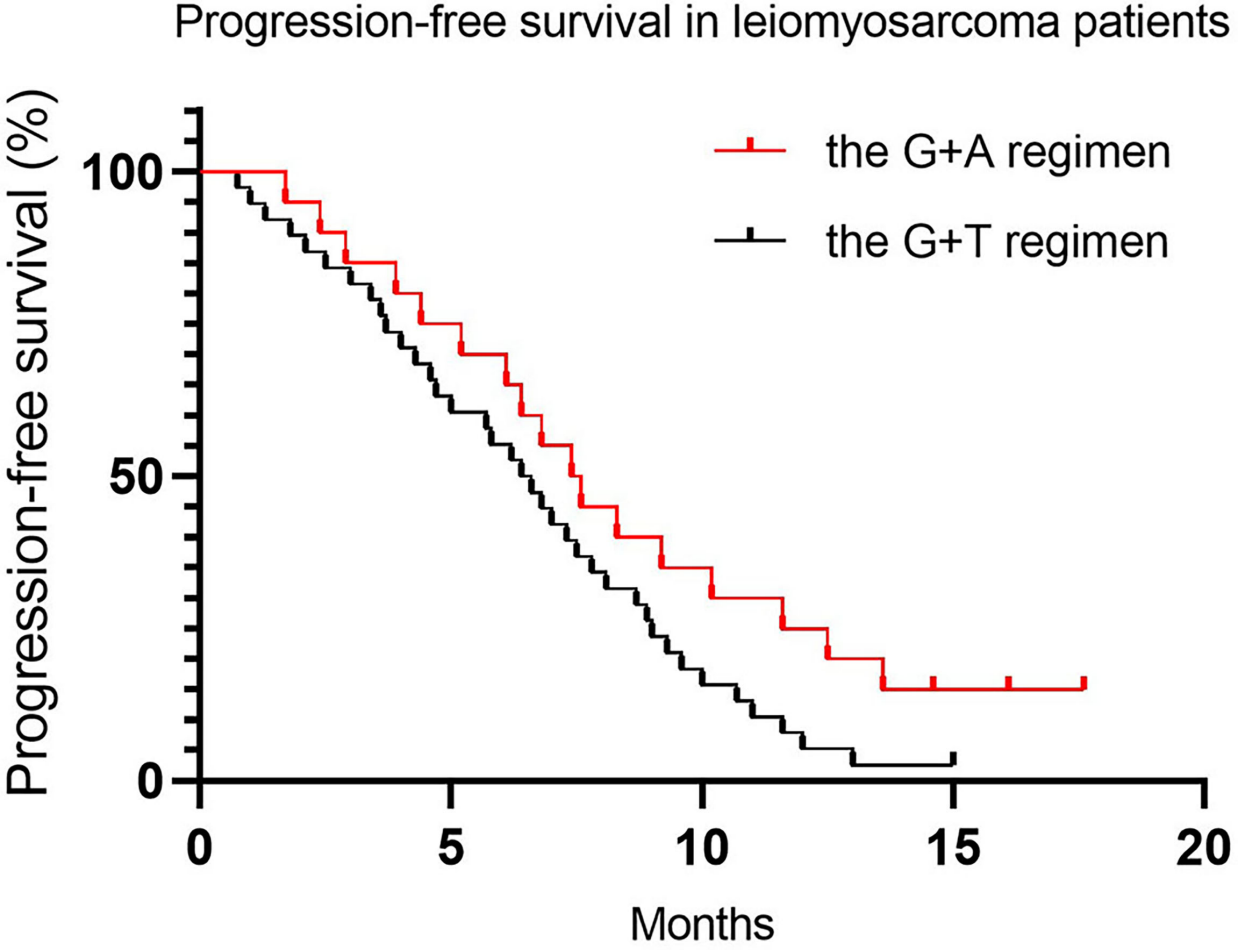
Progression-free survival in leiomyosarcoma patients.

**Figure 4:**
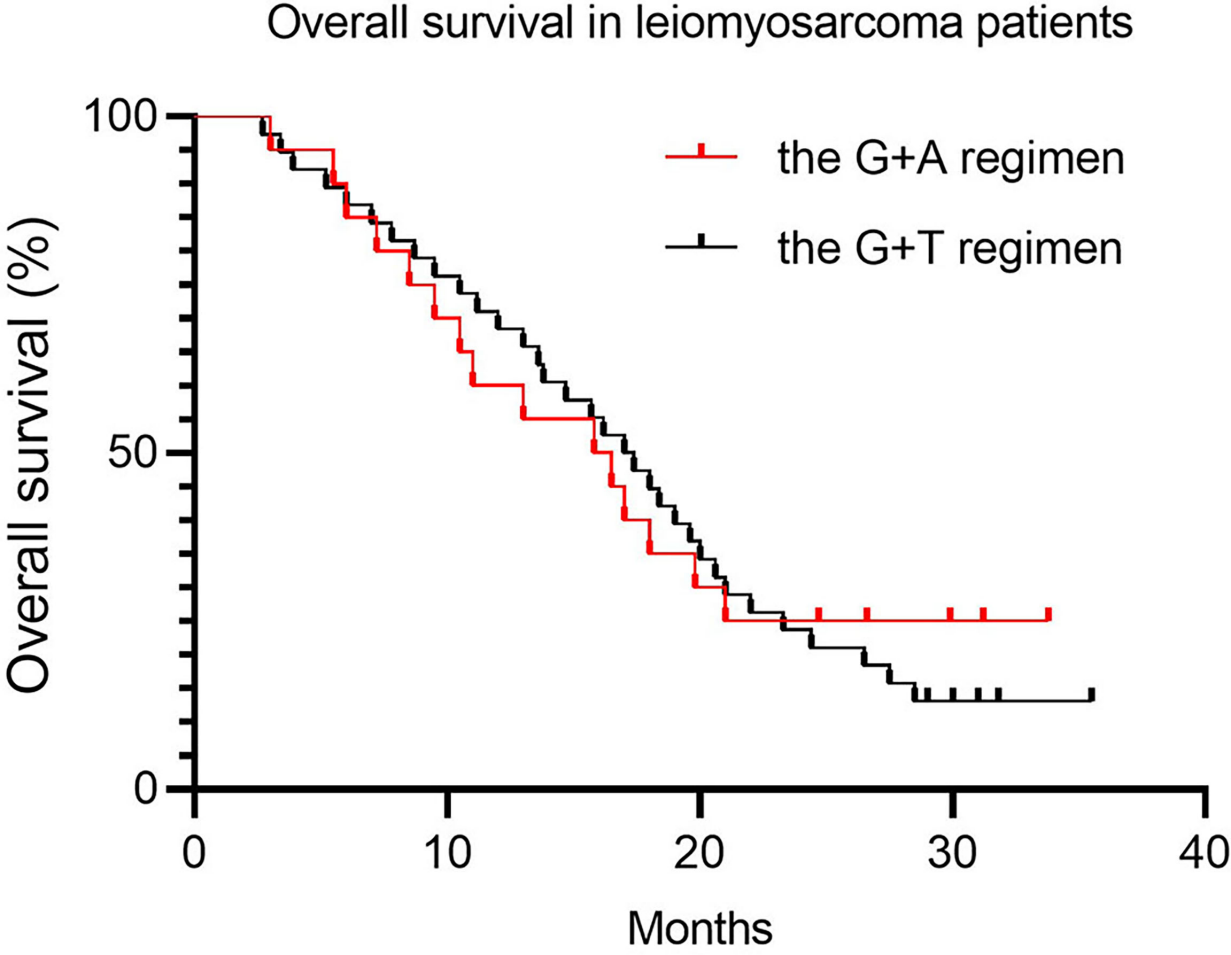
Overall survival in leiomyosarcoma patients.

Since nearly 50% of patients (45.7% and 48.8% for the G+D and G+A groups, respectively) were diagnosed with locally advanced or metastatic LMS, we conducted a separate analysis to compare whether there were significant differences between LMS patients treated with the two regimens. There was no significant statistical difference in PFS and OS, with a median PFS of 6.2 months (95% CI 5.3–8.2) in the G+D group and 7.1 months (95% CI 5.9–9.9) in the G+A group (**Table 5** and **Figure 3**, *p* = 0.08), and a median OS of 15.7 months (95% CI 12.7–18.5) and 13.6 months (95% CI 10.5–17.9) in the G+D and G+A groups, respectively (**Table 5** and **Figure 4**, *p* = 0.76). There were too few patients with other histological subtypes to make a meaningful comparison of results between the two groups.

**Table 5:** Efficacy of patients with leiomyosarcoma and treated as first-line treatment.

PFS and OS	G+D (Median, 95% CI) (months)	G+A (Median, 95% CI) (months)	*p*-Value
**Leiomyosarcoma PFS**	6.5 (5.4–7.7)	7.5 (6.2–10.6)	0.08
**Leiomyosarcoma OS**	17.2 (14.4–20.1)	16.2 (12.1–20.7)	0.76
**First-line PFS**	6.2 (5.3–8.2)	7.1 (5.9–9.9)	0.51
**First-line OS**	15.7 (12.7–18.5)	13.6 (10.5–17.9)	0.62

95% CI, 95% confidence interval; PFS, progression-free survival; OS, overall survival; G, gemcitabine; D, docetaxel.

**Figure 5:**
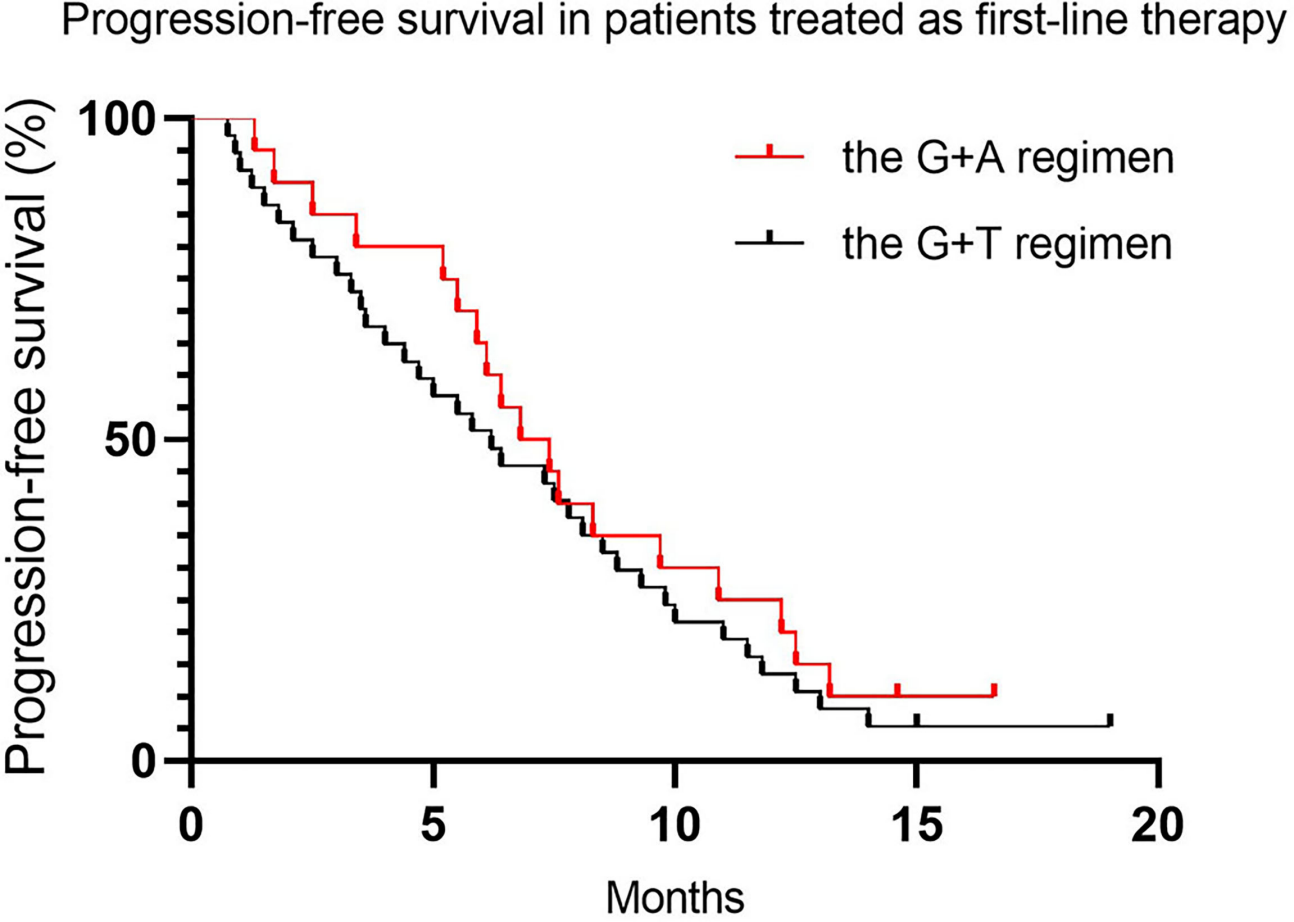
Progression-free survival in patients treated as first-line therapy.

**Figure 6:**
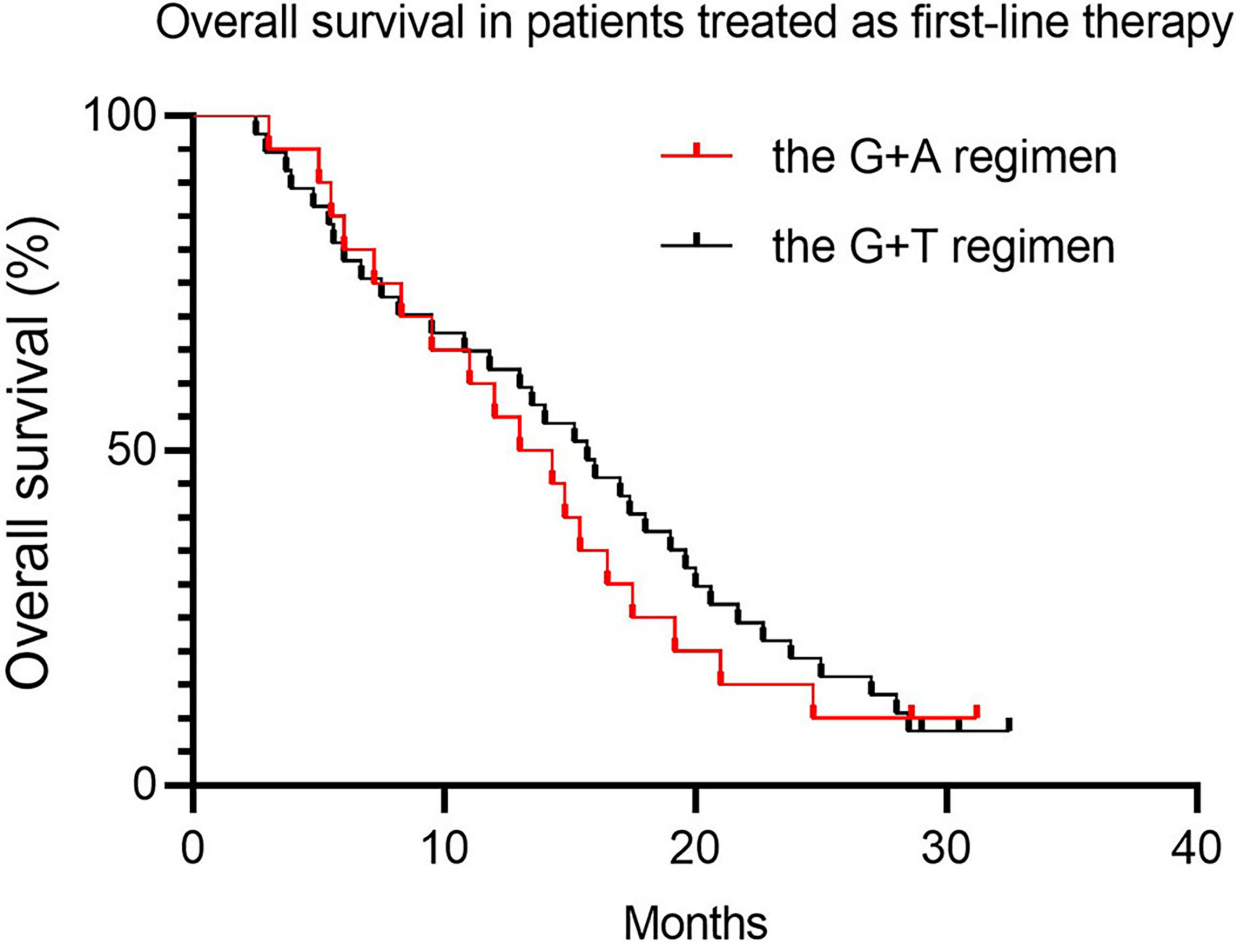
Overall survival in patients treated as first-line therapy.

Approximately 50% of patients (45.7% and 48.8% for the G+D and G+A groups, respectively) received either G+D or G+A as first-line therapy (more than 1 year after adjuvant therapy). We conducted an exploratory analysis to compare the clinical outcomes between patients treated with the two regimens as first-line treatment. The PFS was not a statistically significant difference, with a median PFS of 6.5 months (95% CI 5.4–7.7) vs. 7.5 months (95% CI 6.2–10.6) for the G+D and G+A groups, respectively. (**Table 5** and **Figure 5**, *p* = 0.51). The OS was similar, with a median OS of 17.2 months (95% CI 14.4–20.1) vs. 16.2 months (95% CI 12.1–20.7) for the G+D and G+A groups, respectively (**Table 5**, and **Figure 6**, *p* = 0.62).

### Toxicity

Both G+D and G+A were generally well tolerated. The grade 3/4 AEs are displayed in **Table 6**. The most frequent grade 3/4 AEs in the G+D and G+A groups were neutropenia (48% vs. 34%), thrombocytopenia (35% vs. 34%), anemia (25% vs. 12%), and transaminase elevation (15% vs. 17%). Although most types of AEs were consistent, the proportion of patients who experienced grade ≥3 AEs was higher in the G+D group than that in the G+A group (68% vs. 44%), especially hematological AEs observed in 45 (56%) vs. 14 (34%) patients in the G+D and G+A groups, respectively. It is worth noting that three patients experienced grade ≥3 pneumothorax events. The majority of the reasons for dose delay and dose reduction were febrile neutropenia, other hematological toxicities, and non-hematological toxicities. No deaths related to drugs have been reported.

**Table 6:** Toxicity.

Grade 3/4 adverse events (*n* = 122)	Patients, *n* (%)		*p*-Value
	Gemcitabine+Docetaxel (*n* = 81)	Gemcitabine+Anlotinib (*n* = 41)	
**ALL**	55 (68)	18 (44)	<0.01
**Hematological AEs**	45 (56)	14 (34)	0.03
** Neutropenia**	39 (48)	14 (34)	0.14
** Febrile neutropenia**	15 (19)	3 (7)	0.10
** Anemia**	20 (25)	5 (12)	0.10
** Thrombocytopenia**	28 (35)	14 (34)	0.96
**Non-hematological AEs**	30 (37)	13 (32)	0.32
** Fatigue and muscle soreness**	7 (9)	2 (5)	0.72
** Diarrhea**	5 (6)	2 (5)	1
** Stomatitis**	2 (2)	1 (2)	1
** Vomiting**	5 (6)	2 (5)	1
** Transaminase elevation**	12 (15)	7 (17)	0.75
** Elevated bilirubin**	2 (2)	4 (10)	0.19
** Pneumothorax**	1 (1)	3 (7)	0.21
** Hypertension**	4 (5)	6 (15)	0.14
** Edema**	4 (5)	0	NA
** Thromboembolic**	2 (2)	1 (2)	1

## Discussion

In this retrospective study, we collected and compared the efficacy and toxicity of G+D and G+A in patients with advanced STS; 122 patients (81 patients receiving G+D and 41 receiving G+A) were included. Patients’ characteristics of the two groups were observed to be well balanced at baseline. G+D and G+A showed similar clinical efficacy (ORR: 15% and 14%, median PFS: 6.0 and 7.0 months, and median OS: 9.8 and 11.2 months, respectively), whereas G+A had a lower proportion of patients who experienced grade ≥3 AEs compared to G+D. These results suggest that G+A may be a novel treatment candidate for advanced STS, with similar efficacy to G+D but less toxicity.

The chemotherapy options for advanced STS are limited. The standard chemotherapy regimen is doxorubicin and/or ifosfamide. In recent decades, several exploratory studies (26–28) have shown that doxorubicin combined with other antineoplastic agents, in particular with the alkylating agent, may increase response rates or/and median PFS with increased AEs but fail to improve OS; although olaratumab (a PDGFR inhibitor) plus doxorubicin had a greater median OS than doxorubicin alone in advanced STS in the Phase IB/II study (29) (26.5 months vs. 14.7 months). Regrettably, the subsequent Phase III study including more similar patients did not provide an OS benefit (30) (20.4 months vs. 19.7 months). Since Hensley and colleagues (14) reported a response rate of 55% and a median PFS of 6.5 months in advanced LMS (uterus and others), the patients were given G 900 mg/m^2^ on days 1 and 8 and D 100 mg/m^2^ on day 8 for a 21-day cycle. G+D has been widely used in advanced LMS and STS with promising efficacy. In the first-line treatment setting, the GeDDiS study (31) showed that G+D (G 675 mg/m^2^ on days 1 and 8 and D 75 mg/m^2^ on day 8 every 3 weeks) achieved similar antitumor activity to doxorubicin (doxorubicin 75 mg/m^2^ on day 1 every 3 weeks) in patients with unselected subtypes of STS, with a median PFS of 23.7 weeks and 23.3 weeks, and an ORR of 19% and 20%, respectively. However, G+D had higher costs, was more difficult to deliver, required more frequent and longer hospital visits, and caused more non-hematological toxicities than doxorubicin. With the failure of an anthracycline-based regimen setting, several studies compared the efficacy and toxicity of G+D versus G monotherapy in advanced sarcomas. In 2012, the TAXOGEM study (16) showed that G alone (G 1,000 mg/m^2^ on days 1, 8, and 15 every 4 weeks) and G+D (G 900 mg/m^2^ on days 1 and 8 and D 100 mg/m^2^ on day 8 every 3 weeks) yielded an ORR of 19% and 24% in patients with uterine LMS, with a median PFS of 5.5 and 4.7 months, and an ORR of 14% and 5% in patients with non-uterine LMS, with a median PFS of 6.3 and 3.8 months, respectively. Meanwhile, a Phase II study (32) published by Maki showed that G+D yielded greater median PFS and OS than G alone in metastatic STS, but increased toxicity. These results suggest that both G monotherapy and G+D may be an option for advanced STS.

Our study showed that either the median PFS of 5.8 months for G+D or the median PFS of 6.5 months for G+A may be higher than those reported by Neeta Somaiah (33), but consistent with several previous reports including the GeDDis study (31), the LMS03 study (34), and Hensley’s study (14). The different clinical outcomes may be due to differences in the initial dose, dose intensity and infusion rate of drugs, as well as patients’ characteristics at baseline. In a randomized Phase II trial reported by Neeta Somaiah in 2021 including 90 patients with advanced STS who were randomized, 1:1 received either gemcitabine plus pazopanib (G+P) or gemcitabine plus docetaxel (G+D). Both G+D and G+P showed a median PFS of 4.1 months with similar toxicity. There may be many reasons why Neeta Somaiah’s research differed from ours. Firstly, a larger proportion of advanced LMS patients were included in the present study (47.5% vs. 31%) than in Somaiah’s study. Several studies (35, 36) have shown that LMS may be superior to non-LMS in chemotherapy sensitivity, with a greater response rate, and median PFS and OS. However, the results of the subgroup analysis of our study reveal that no significant differences were observed in ORR and median PFS between LMS and other subtypes. It should be noted that a large proportion of histopathological subtypes (LMS for 47.5%, UPS for 13.9%, and LPS for 13.1%) may be considered to be chemosensitive to gemcitabine-based regimen in this study. Secondly, patients in our study experienced fewer dose reductions than Neeta Somaiah ‘s study (24% vs. 58%) due to toxicity. In Neeta Somaiah’s study, 58% of patients in the G+D group underwent dose reduction and 80% of patients underwent dose reduction in the G+P group, while in our study, 30% of patients in the G+D group had dose reduction and 20% of patients in the G+A group underwent dose reduction, which may have an impact on efficacy. It is worth noting that only 20% of patients in the G+A group underwent dose reduction, significantly less than previously reported, which may be related to the fact that anlotinib avoided overlap with gemcitabine in this study and that there is little toxicity from anlotinib itself. According to the toxicities of targeted agents reported in previous studies (10, 37), the rate of grade ≥3 adverse reactions of anlotinib was significantly lower than that of pazopanib. Thirdly, there is a higher proportion of patients receiving the regimens as the first-line therapy (46.7% vs. 18%) in our study compared to Neeta Somaiah ‘s study. The high proportion of patients in this study is due to aggressive neoadjuvant chemotherapy based on anthracyclines in China. When the tumor recurred more than a year later, they were treated with G+D or G+A as first-line therapy. The efficacy of the present study is similar to that of the GeDDis study, with an ORR of 20% and a median PFS of 23.7 weeks.

Overall, the two treatment regimens were well tolerated, and the majority of AEs were consistent with previous studies and no new AEs were observed. The common grade 3/4 AEs of G+D were neutropenia, thrombocytopenia, anemia, and febrile neutropenia, and the common grade 3/4 AEs of G+A were neutropenia, thrombocytopenia, transaminase elevation, and hypertension. Edema was mainly related to docetaxel, and fatigue and muscle soreness may be caused by long-term use of G+D, while pneumothorax and hypertension were mainly related to anlotinib. The proportion of patients with grade ≥3 AEs was higher in the G+D group than in the G+P group, and the major differential AEs were hematological AEs. Although there is no comparable study of docetaxel versus anlotinib in advanced STS, the rate of grade ≥3 hematological toxicity related to anlotinib reported by Chi (37) was lower than that related to docetaxel reported by van Hoesel (38) (0% vs. 89%). Thirty percent of patients in the G+D group needed at least one dose reduction due to adverse reactions, while only 20% did in the G+A group.

Palliative systemic treatment of advanced STS aims to reduce the tumor burden, thereby reducing symptoms and improving quality of life. To avoid primary resistance and acquired resistance to the tumor, as well as prolong the time of systemic therapy control, aggressive systemic therapy without significantly increased toxicity has become a treatment option. In this study, we explored the clinical efficacy and safety of G+A as a novel therapy for advanced STS, and the results show that G+A achieved similar efficacy to G+D with less toxicity, suggesting that G+A could be used as an alternative to G+D, and the therapy might be suitable for patients with poor physical quality and intolerance to G+D or even the standard treatment. Both gemcitabine plus anti-tumor drugs and gemcitabine plus docetaxel combined with other anti-tumor drugs have been explored in advanced STS. Although few positive results have been reported in terms of efficacy, the results of some studies provide a reference for future studies. The LMS03 study (34) assessed the efficacy and safety of G plus pazopanib followed by pazopanib (G 1,000 mg/m^2^ on days 1 and 8 and pazopanib 800 mg daily of each for 21 days, for no more than 8 cycles, followed by pazopanib) as the second-line treatment in advanced LMS; although the PFS rate at 9 months (PFR 9m) of 32% with a median PFS of 6.5 months failed to meet the target of 44%, a PFR 9m of 34.6% and a median PFS of 7.1 months in the per-protocol population were promising. The PAPAGEMO study (39) examined the efficacy of pazopanib plus G (pazopanib 800 mg once daily and G 1,000 mg/m^2^ on days 1 and 8 every 3 weeks) and G (G 1,000 mg/m^2^ on days 1 and 8 every 3 weeks) in advanced STS patients who failed with anthracycline and/or ifosfamide. The results show that compared with pazopanib alone, G+D significantly increased PFSR at 12 weeks (74% vs. 47%), with prolonged median PFS (5.6 months vs. 2.0 months), respectively. Interestingly, in a Phase III study (40), including 90 LMS patients, which evaluated the efficacy of G+D with or without bevacizumab as a first-line treatment for metastatic uterine LMS, the ORR was 31.5% and median PFS was 6.2 months in the G+D group, while the addition of bevacizumab to G+D did not improve the efficacy with an ORR of 35.8% and a median PFS of 4.2 months. These data suggest that although some antiangiogenic agents, particularly bevacizumab, may achieve a moderate antitumor activity in advanced STS, these agents may not be suitable for combination with chemotherapy.

The limitations of this study include the following: First, due to the retrospective and non-randomized nature of this study, documented toxicity may be incomplete. However, grade ≥3 AEs would be evaluated carefully and thoroughly, as such observations may contribute to clinical decision-making. Second, anlotinib is considered to inhibit the proliferation of pericytes and result in impaired tumor angiogenesis mainly through an EGFR2 signaling pathway. EGFR2 expression of the tumor and the relationship between EGFR2 and prognosis were not identified in this study. Third, due to the low number of non-LMS sarcomas, it was impossible to conduct a meaningful comparative analysis of the specific subtypes between the two groups.

## Conclusion

In summary, our study shows that the G+A regimen obtains modest ORR, and median PFS and OS, similar to the G+D regimen, but less toxic. The prognosis of advanced STS remains poor, new therapeutic strategies need to be explored, and this study may serve as a benchmark for such trials in the future.

## Data Availability Statement

The original contributions presented in the study are included in the article/supplementary material. Further inquiries can be directed to the corresponding author.

## Ethics Statement

The studies involving human participants were reviewed and approved by Henan Cancer Hospital. The patients/participants provided their written informed consent to participate in this study.

## Author Contributions

All authors listed have made a substantial, direct, and intellectual contribution to the work, and approved it for publication.

## Conflict of Interest

The authors declare that the research was conducted in the absence of any commercial or financial relationships that could be construed as a potential conflict of interest.

## Publisher’s Note

All claims expressed in this article are solely those of the authors and do not necessarily represent those of their affiliated organizations, or those of the publisher, the editors and the reviewers. Any product that may be evaluated in this article, or claim that may be made by its manufacturer, is not guaranteed or endorsed by the publisher.
